# New Developments and Possibilities in Reanalysis and Reinterpretation of Whole Exome Sequencing Datasets for Unsolved Rare Diseases Using Machine Learning Approaches

**DOI:** 10.3390/ijms23126792

**Published:** 2022-06-18

**Authors:** Samarth Thonta Setty, Marie-Pier Scott-Boyer, Tania Cuppens, Arnaud Droit

**Affiliations:** Molecular Medicine Department, CHU de Quebec Research Center-UL, Quebec City, QC G1V 4G2, Canada; samarth.thonta-setty@crchudequebec.ulaval.ca (S.T.S.); mariepier.scottboyer@crchudequebec.ulaval.ca (M.-P.S.-B.); tania.cuppens@crchudequebec.ulaval.ca (T.C.)

**Keywords:** rare diseases, machine learning, reanalysis

## Abstract

Rare diseases impact the lives of 300 million people in the world. Rapid advances in bioinformatics and genomic technologies have enabled the discovery of causes of 20–30% of rare diseases. However, most rare diseases have remained as unsolved enigmas to date. Newer tools and availability of high throughput sequencing data have enabled the reanalysis of previously undiagnosed patients. In this review, we have systematically compiled the latest developments in the discovery of the genetic causes of rare diseases using machine learning methods. Importantly, we have detailed methods available to reanalyze existing whole exome sequencing data of unsolved rare diseases. We have identified different reanalysis methodologies to solve problems associated with sequence alterations/mutations, variation re-annotation, protein stability, splice isoform malfunctions and oligogenic analysis. In addition, we give an overview of new developments in the field of rare disease research using whole genome sequencing data and other omics.

## 1. Introduction

A rare disease (RD) is defined as a condition that affects fewer than 1 in 2000 people [1]. Overall, it is estimated that there are around 8000 rare diseases that impact the lives of around 300 million people in the world [2]. It is important to consider that current standard clinical diagnostic practices can take a long time to diagnose rare diseases, and in some cases, up to 30 years [3]. Approximately 80% of RDs are believed to have a genetic cause [4]. The rapid advances in genomic technologies and bioinformatics analysis have enabled the discovery of genetic causes of 20–30% of rare diseases [3] using high-throughput sequencing (HTS) of the whole exome. A study showed that HTS technologies have enabled a ~40% diagnostic rate compared to ~10% using traditional methodologies [3]. It is to note that for monogenic diseases, genetic causes have been implicated in only 30–40% of the diseases [5].

Therefore, recent pipelines that have targeted rare disease discovery have included new analysis strategies such as the NHS (National Health Service) study on RD [6]. The input of these pipelines are raw reads derived from sequencing the whole exome (called whole exome sequencing/WES) and other technologies including hole genome sequencing (sequencing the entirety of the genome/WGS), RNA-seq (sequencing the RNA pool of a tissue/group of cells), targeted-seq (sequencing a targeted region of the genome or exome), etc., with each having their own advantages and drawbacks. While WES has been responsible for most gene discoveries through HTS, whole genome sequencing (WGS) is superior in detecting copy number variants, chromosomal rearrangements and repeat-rich regions. Additionally, targeted panels are commonly used for diagnostic purposes as they are extremely cost-effective and generate manageable quantities of data, with no risk of unexpected findings. However, in instances of diagnostic uncertainty, it can be challenging to choose the right panel, and in these circumstances, WES has a higher diagnostic yield [7]. Moreover, depending on the rare disease context, reanalysis of WES-derived genetic variants can sometimes improve diagnostic yields [5] or result in the downgrading of the pathogenicity status of some previously reported variants [8]. This leads to frequent updating of the variant databases (DB) and supports the importance of data reanalysis.

Although the diagnostic rate has improved due to HTS, because of these challenges, there are vast troves of underexplored genomic datasets, leading to an expensive non-diagnosis and lack of actionable insights for patients. Therefore, more efforts are being made to solve previously unsolved rare diseases by reanalyzing previously generated sequencing data using new methodologies [9,10]. One of the first reanalysis studies showed an increase in diagnostic yield by 18% (absolute diagnostic yield increased from 25.4 to 31.4%) [11], indicating the possibility of gathering new insights into the underpinnings of rare diseases.

As the amount and complexity of genomic data increases, researchers are turning to artificial intelligence (AI) and machine learning (ML) for the reanalysis of already existing data to answer health care and research questions. ML is a process by which machines can be given the ability to learn from a set of data. For the application to genomics, several domains have been explored to predict from validated data the effect of a mutation/alteration of the genome.

Several papers have shown that the reanalysis of WES data could improve diagnostic rates of patients with rare diseases that could not obtain an initial molecular diagnosis. However, the description of procedures to improve the diagnostic yield for re-analysis has been limited. In this review, we are describing analysis from the simplest (single variant analysis) to more complex (gene–gene interactions) that can be performed on WES data. In this review, we systematically survey the latest developments in the application of machine learning in the discovery of the genetic causes of rare diseases, especially using previously available WES data of unsolved diseases. Currently, machine learning tools have been developed to focus on ameliorating issues dealing with:

1. Variant pathogenicity predictions, where new ML algorithms are used to better predict variant pathogenicity [12,13];
2. Variant re-annotation efforts, which require constant re-annotation or update of variants of uncertain significance [14,15];
3. Splicing isoform alterations, where splice isoforms are altered leading to disease consequences [16];
4. Consequences of sequence alterations, where mutations can lead to rare diseases [17,18];
5. The diagnosis of RD of oligogenic inheritance (for example, digenic inheritance), where multiple genes are responsible for causing rare diseases [19].

In addition, we refer to the new developments in the field of rare disease research using results from WGS data analysis and future AI technologies, including ML technology. The public availability of high throughput sequencing data and emerging ML methods to discover the genetic causes of rare diseases have increased in recent times [20]. Since the arrival of deep learning nets, there has been a rapid need to assess which methods are applicable for rare diseases [21,22].

## 2. Reanalysis Methodologies Using Machine Learning

Recently, AI and ML techniques have been successfully applied to basic research, diagnosis, drug discovery and clinical trials [20,23]. AI has been used in a significant manner in the field of underrepresented and mis/undiagnosed rare diseases [20]. Importantly, AI technologies in combination with data analysis from diverse sources (e.g., multi-omics, phenotypic data, image data, etc.) can be used to overcome the challenges associated with rare diseases such as low diagnostic rates, reduced numbers of patients, geographical dispersion, and lack of funding, leading to better drug development [24]. Presently, there are many AI approaches, including machine learning techniques that are being used in understanding and reanalyzing unsolved RDs and this review aims to collect and summarize such approaches.

The methods presented in the following review pertain to ML methodology such as ensemble ML methods, support vector machines (SVM) and neural networks (NN). In brief, ensemble methods make use of a combination of many simple models to obtain the best predictive models [25], whereas SVMs are a supervised classification approach used to classify samples based on a known feature set defining the classes [26]. Additionally, NNs comprise artificial neurons with weights that learn from data [27]. The emergence of neural network-based tools to filter and identify rare disease variants is promising. In fact, NNs currently result in the least error rates when detecting rare disease variants using genomics and transcriptomics datasets [28].

Furthermore, it has been reported in a systematic review that ensemble methods (36.0%), SVM (32.2%) and artificial NNs (31.8%) were used in publications dealing with ML approaches in RD [20]. Most studies used machine learning for diagnosis (40.8%) or prognosis (38.4%) whereas studies aiming to improve treatment were scarce (4.7%). However, only 26.5% of these studies had genomics and transcriptomics datasets as input. Even among many of these datasets, there were inherent issues in applying ML to rare diseases. For example, patient numbers in the studies were small, typically ranging from 20 to 99 (35.5%) [20], which is a known hindrance for the identification of genetic variants implicated in rare diseases, resulting in small data challenges and low statistical power [29]. Nevertheless, novel statistical approaches have been developed to consider smaller patient sizes and serve as a dataset for modern ML algorithms, specifically designed to help solve rare disease issues [30].

**Figure 1:**
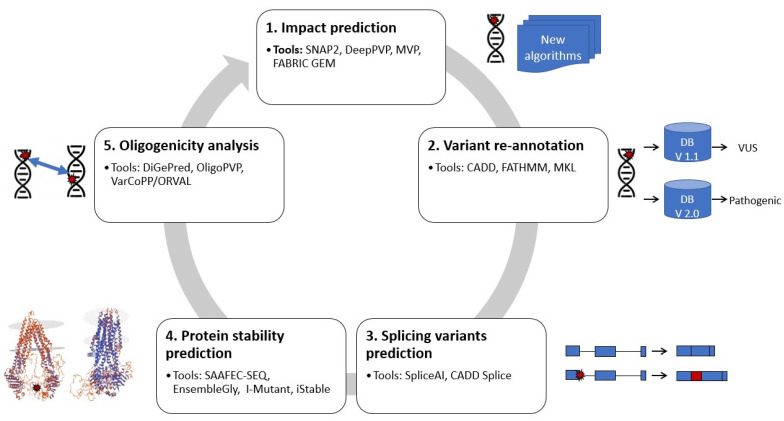
Overview of the machine learning strategies for WES reanalysis from the single variant analysis to more complex genomics event (gene–gene interactions). 1. Predicting the impact of sequence alterations/mutations. This strategy consists of predicting the effect of a sequence change on protein. 2. Variant re-annotation strategies try to re-annotate the variants after availability of new information/discoveries. 3. Variants that alter splice isoform frequencies are predicted using methods in this strategy. 4. In this category, protein folding/protein structural differences are assessed. 5. Oligogenic analysis is a strategy for analysis of digenic (gene pairs) and oligogenic diseases. Examples of tools for reanalysis of rare diseases using machine learning are presented for each strategy.

In the next paragraphs, we will introduce tools that are in use or could be used to help in identifying the causes of unsolved rare diseases (Figure 1). The tools used different ML methodologies with pre-existing WES or WGS datasets to predict the impact of sequence alterations/mutations, variation re-annotation, protein stability, splice isoform malfunctions and oligogenic analysis.

### 2.1. Predicting the Impact of Sequence Alterations/Mutations

Sequence alterations (such as small indels) or mutations in the gene can lead to deleterious effects [17,18]. However, identifying the causative mutations of the rare disease requires annotation using multiple databases and then applying filters based on allele frequencies, pathogenicity scores associated with the variants [14]. Advances in combining information from multiple predictive algorithms, for instance, the use of ensemble tools such as REVEL [31], have led to an increased understanding of the role of missense mutations in causing rare diseases. However, they are not up to mark as they are still not highly concordant with clinically relevant variant lists [32].

A recent study showed the use of statistical analysis to correlate the location of variants and their pathogenicity. The study presented correlations between variant location information with pathogenicity scores for those variants predicted using in silico prediction algorithms such as SNAP2 within the Wolframin gene (*WFS1*) on rare psychiatric disorders [33]. These variants were obtained from a list of published and curated mutations that pertain to psychiatric disorders. This highlights the potential of in silico approaches in re-identifying significant mutations among a bigger list of known rare mutations.

New tools built with deep neural networks have been employed to learn from phenotype information, in conjunction with genomic information of variants. This is the case for the tool DeepPVP, which has been used to identify the causes of different rare diseases [34]. Phenotype information has been shown previously in many publications to help narrow down causal variants [35,36]. The use of important clinically relevant information to add training information such as HPO (human phenotype ontology) to deep neural net models has been shown to improve performance and assist in reducing the effort of clinicians [6] for instance the Rare Disease Auxiliary Diagnosis system (RDAD) [37]. This presents a novel avenue for rediscovery efforts where phenotype information was not used previously.

Additionally, predictive tools such as MVP (missense variant pathogenicity prediction) have been developed for specific kinds of variants (for missense rare variant pathogenicity predictions). This allows the identification of disease-related missense mutations which may not be captured by non-specific tools [38]. MVP makes use of a deep residual network to gain insights from large training data sets consisting of both genes that are intolerant of loss of function variants and those that are tolerant to effectively delineate their effects.

Finally, big consortia such as the National Health Service, England [6] have been using FABRIC GEM, an NN based prioritization tool that vastly improves the detection of causal genes and variants related to unsolved rare diseases. FABRIC GEM works as a complete variant prioritization platform and has been shown to perform better than other solutions such as VAAST [39], Phevor [40] and Exomizer [41]. It has also sped up the interpretation by reducing the time taken to clinically review pathogenic variants within genes by reducing the number of genes in review to an average of just two genes per case instead of tens of genes in the case of competing tools [9,42].

### 2.2. Variant Re-Annotation

Both protein-coding and rare disease-associated variants have been discovered through the analysis of exome sequencing data [43]. However, these variants need to be properly annotated to help interpret possible functional mechanisms linking them with rare diseases of interest.

The American College of Medical Genetics and Genomics-Association for Molecular Pathology (ACMG-AMP) guidelines have provided a common framework for variant classification [44]. Even though the framework provides a way to bin rare variants into multiple categories such as variants of uncertain significance (VUS) or benign, it is important to periodically recalibrate or re-classify them according to novel discoveries or changing landscapes in variant biology [45]. In this regard, there have been ML-based efforts to identify and assign the pathogenicity of variants in rare diseases [6,46].

A commonly used ML algorithm to detect causal variants of rare diseases is called SVM. The tools that employ SVM are usually dealing with the annotation of variants using previously available or newly updated features delineating a disease-related genetic variant. The putative disease-related SNP predictive tools called CADD [13] and Fathmm-MKL [47] have been used in the RD community for many years to predict and attribute the pathogenicity/disease relevance of genetic variants. Once a discovery is made, the variants must be continuously re-annotated to score and classify the variants of interest in regular update cycles. This allows the classification of the VUS to be annotated either as a harmful or pathogenic variant according to current developments [45]. Although, it has been observed that delineating variant significance is highly influenced by thresholds and context [48]. A recent study using a rules-based algorithmic approach showed that 125 VUS were reclassified in 114 unsolved rare inherited retinal dystrophy patients which helped in the diagnosis of the disease. It was shown using validation datasets that ~70% of VUS in these patients were reclassified as pathogenic [49].

Meta-SVM employs a meta-analysis method to compile many OMICs datasets such as breast cancer expression profiles provided by The Cancer Genome Atlas (TCGA) including mRNA, copy number variation (CNV) and epigenetic DNA methylation to discover understudied genetic variants in rare TCGA datasets [50]. This could be extended to rare diseases where multiple omics datasets are available to identify features such as gene sets that go haywire in diseases regulated by intersecting pathways. However, there have been instances where META-SVM has been shown to be a poor predictor of protein function when compared to published annotated databases that predict non-pathogenic variants as pathogenic, and vice versa [48].

### 2.3. Predicting Splicing Variants

Variants that affect splicing are significant contributors to rare diseases, but they are often overlooked. This observation can be in part explained by the fact that very often during variant analysis synonymous variants are ignored because they have no impact on the final protein sequence [51].

SpliceAI has been used to understand RDs with intellectual disability and autism spectrum disorders. The tool makes use of deep residual NNs to identify splice-relevant mutations, or mutations that affect splicing and result in aberrant isoforms, thereby causing the dysfunction in patients with rare diseases. SpliceAI has already been used to infer the splicing effects of mutations that have been missed from previous databases [52]. This presents a compelling case for reanalysis of RDs as splicing defects are implicated in 15–50% of human diseases and are frequently overlooked in rare disease diagnosis [53]. In fact, SpliceAI shows high accuracy in predicting splicing-related mutations that affect function (>90%) [53].

CADD-Splice is a recent splicing tool predicting variant effects on splicing using deep neural networks (DNNs) as an addition to the CADD variant pathogenicity prediction tool. CADD-Splice integrates splice tools including MMsplice [54] and SpliceAI to predict variants that highly alter normal splicing patterns in disease [55].

### 2.4. Predicting Protein Stability

In genetic diseases, abnormal protein stability typically results from mutations that alter the amino acid sequence of proteins. Protein stability can be defined as a balance of forces that determine whether a protein will be in its native folded conformation or in a denatured state (unfolded or extended). Glycosylation is one of the most common forms of post-translational modification. Several studies have shown that it alters not only the thermodynamic stability but also the structural characteristics of folded proteins by modulating their interactions and functions. Their inhibition and disruption have been implicated in diseases ranging from diabetes to degenerative disorders [56]. In certain rare diseases, misfolded proteins can be retained in the endoplasmic reticulum (ER), in which case they do not reach sites in the cell where they are normally active, resulting in disease [57]. Based on this information, several tools have been developed to predict protein stability, glycosylation and misfolding.

The tool SAAFEC-SEQ (single amino acid folding free energy changes-SEQ) is based on the pseudo-position specific scoring matrix (PsePSSM) algorithm to predict thermodynamic stability changes from a single mutation in a protein [58]. SAAFEC-SEQ combines physicochemical properties, sequence characteristics and evolutionary information to calculate the change in stability-free energy that a mutation causes. EnsembleGly compiles many ensembles of SVM to help identify variants of interest in glycosylation-related disorders [59]. SVMs have also been employed within I-Mutant [60] and iStable [61] to deduce the causal variants of the RD Mevalonic kinase deficiency [62,63]. Although not recently developed tools, these are specifically related to specific protein residue modifications that might have been overlooked by those interested in other directions of research. A reanalysis using these kinds of tools might be beneficial in screening potential protein alterations.

### 2.5. Oligogenicity Analysis

Contrary to monogenic traits, oligogenic traits are produced by the interaction of genes at many loci. For example, digenic inheritance is a mechanism whereby the interaction between two genes is required for the expression of a phenotype or a disease. Digenic inheritance and therefore the analysis of gene pairs could be a key mechanism to better understand rare diseases [64].

DiGePred, a random forest classifier, has been developed to specifically identify candidate disease gene pairs (digenic diseases) by features derived from biological networks, genomics, evolutionary history and functional annotations [65]. DiGePred used an ML strategy called ensemble method which has been used in RD classification and is based on random forest classifiers where multiple weak decision trees are combined to generate a better predictive outcome in terms of classification [20]. The use of DiGePred has helped in the discovery of genetic causes for rare non-monogenic diseases by providing a score to evaluate variant gene pairs for the potential to cause digenic disease [65]. This type of analysis could be then used to assess the prevalence of putative gene pairs in undiagnosed rare non-monogenic diseases. The advantages of such a predictive system lie in the identification of neglected digenic disorders, incorrectly classified as monogenic rare diseases. If a disease presents variant gene pairs and is unsolved, such a tool might be of effective use.

Recent studies have also focused on developing tools to prioritize the oligogenic variants that are responsible for rare diseases. Here, we discuss two important tools which make use of the DIgenic diseases DAtabase (DIDA) [66] as input training data, albeit with a small training sample size, for pathogenicity predictions. Firstly, OligoPVP is a tool that combines an RF classifier and a deep neural net to predict variant pathogenicity of a combination of oligogenic disorders, using a feature set from different tools such as CADD, DANN to classify those variants as causative or non-causative. Furthermore, VarCoPP [67] is a more recent tool that also uses an RF classifier to classify oligogenic variants. The VarCoPP classifier algorithm makes use of 11 different biological features compiled by feature importance scores and generates classification scores for paired allelles. Moreover, ORVAL, another tool that extends the use of VarCopp predictions to include more features such as web-based exploration, has been recently used in understanding the pathogenicity of variant combinations within *BBS* gene that are detrimental in non-obese juvenile-onset syndromic diabetic patients [68]. However, these tools are limited by the number of variants that can be studied and require further research [67].

## 3. Emerging Technologies and Methodologies for Reanalyzing Rare Diseases

New emerging technologies such as whole-genome sequencing could be used in the field of rare disease research. In this section we will discuss the potential of WGS and new sequencing technologies, structural variants and multi-omics integration for the reanalysis of rare diseases.

### 3.1. Whole Genome Sequencing and New Sequencing Technologies for Rare Diseases Diagnostics

A recent study has shown that WGS, in combination with clinical data gathered in the 100,000 Genomes Project, has been successfully used to diagnose previously undiagnosed patients with suspected rare diseases [6]. Of the diagnoses that were made, 14% were based on variants found in parts of the genome that would have been missed by other types of tests, such as gene panels or exome sequencing. However, variants were overwhelmingly observed in the coding regions of the genome [69].

In the past few years, sequencing technologies such as single molecule sequencing allow the sequencing of long reads. Single molecule sequencing is a third-generation sequencing technology that helps decode the sequence of a single molecule without any amplification required as in short read NGS technologies. Currently the single-molecule real-time (SMRT) sequencing by Pacific Biosciences (PacBio, Menlo Park, CA, USA), and nanopore sequencing by Oxford Nanopore Technologies (ONT, Oxford, UK) have matured enough to provide sufficiently accurate long reads with read lengths of 1–100 kbp. Single molecule sequencing has allowed an increased resolution of the genome and helped resolve many challenges in the genomics space [70,71]. New tools such as DeepSEA minion have been developed, which make use of unsupervised NNs to learn from MinION sequencing datasets [72]. Although not seen in widespread use as short-read sequencing, single molecule sequencing allows the detection of repetitive regions confidently in clinical diagnosis of diseases [71]. Thus, a combination of next generation sequencing technologies can help in reanalyzing patients with undiagnosed rare diseases.

Additionally, single cell sequencing, which allows the sequencing of each cell type, has matured in recent days. However, single cell sequencing also requires better algorithms and computational power to analyze large datasets with much higher dimensions than non-single cell approaches. The use of deep learning approaches such as autoencoder algorithms has been shown to be quite effective in understanding important insights in cell biology [73,74,75]. There has already been an excellent scoping review here which covers the full scope of these approaches [76]. We have not been able to confirm autoencoder-specific approaches in reanalysis of rare disease variants to date. An avenue of potential future research would be to reanalyze undiagnosed patients with rare diseases using single cell sequencing methods such as scRNA sequencing (the sequencing of the RNA in individual cells) to decipher different cell classes with altered splice isoforms responsible for disease [77].

### 3.2. Structural Variants Analysis

Increased effort is being devoted to the interpretation of structural variants (SVs), which include copy number variants, chromosomal rearrangements and repeat-rich regions [78]. Indeed, array-based comparative genomic hybridization tests yield a ~12% diagnostic rate, with ~8% of patients having CNVs of unknown significance [79]. It should also be mentioned that the development of tools for the detection of all chromosomal rearrangements has developed a lot since this past year and increased effort is made to also perform this on WES data [80]. While individual CNVs are rare, most are frequent and represent a significant and non-rare source of genetic variation in the human genome [81]. It is therefore normal to see an increasing development of ML and AI tools to predict the effect of CNVs as it has been accomplished for SNVs. Several tools, such as StrVCTVRE, promise better annotation, classification and prioritization of SV [82,83,84]. Reanalysis and reinterpretation of unresolved rare disease data including CNV analysis would certainly allow an increase in the diagnosis rate.

### 3.3. Multi-Omics Analysis and Integration

The development of omics technologies (such as epigenomics, transcriptomics, proteomics and metabolomics) can complement ML based approaches in adding molecular insight to genomics datasets.

For example, a recent review by Schlieben et al. [85] has highlighted how RNA sequencing methods can improve the diagnosis of rare diseases. Furthermore, machine learning models can also be used on transcriptome to improve knowledge of rare diseases. One promising model is transfer learning. Transfer learning is an ML technique that repurposes a trained model for one task on a new task. Recently, transfer learning strategies have been used in tools such as MultiPLIER [86] for studying rare diseases. This tool used trained ML models on large transcriptomics datasets and transferred this model to smaller rare disease datasets. This type of ML is a good example of the reuse of transcriptomics datasets to study rare diseases where too few samples are available to have a performing model. The identification of pathobiological mechanisms of rare diseases at various levels of biological organization could also improve our knowledge on rare diseases [87]. Several techniques of multi-omics integration using ML have been developed to better understand how the different omic layers act together. A recent review has shown how these methods have been applied to mitochondrial diseases [88]. Furthermore, a network-based framework could deepen our understanding of disease-associated perturbations of molecular networks. A molecular network can provide insights into complex systems and can reveal informative patterns through the integration of biological omics data. For example, the tool DIGNiFI (disease causing gene finder) and vertex-similarity (VS) have used protein–protein interaction networks to analyze GWAS hits and better understand the mechanism underlying rare diseases [89,90].

## 4. Conclusions

It is important to note that an essential element of reanalysis is data sharing and, therefore, to increase efforts on the reanalysis of existing NGS datasets and improve resolving the causes of rare diseases, researchers and consortia should adhere to the FAIR (findable, accessible, interoperable and reusable) principle of data sharing [91]. The advent of NGS has increased the identification of variants causing rare disease, but even if a variant is not found, it does not mean that the information does not lie within this data. Currently, it is very difficult to verify the impact of all the variants of an individual in a biological way and thus to define with confidence which one is involved in a rare disease. Therefore, many rare diseases remain undiagnosed. The development of new predictive tools is therefore essential to allow the reduction, filtration and prioritization of these variants to facilitate the diagnosis of patients suffering from diseases and more particularly rare diseases.

The tools presented in this review offer many possibilities in the reanalyses of NGS datasets to increase the known information for a variant of concern. The more knowledge there is about the impact of a variant on protein conformation, splicing and even RNA/protein interactions, the better the identification and interpretation of disease-causing variants.

## References

[1] About Cord|Canadian Organization for Rare Disorders. https://www.raredisorders.ca/about-cord/.

[2] Groft S.C., Posada M., Taruscio D. (2021). Progress, challenges and global approaches to rare diseases. Acta Paediatr..

[3] Sawyer S.L., Hartley T., Dyment D.A., Beaulieu C.L., Schwartzentruber J., Smith A., Bedford H.M., Bernard G., Bernier F.P., Brais B. (2016). Boycott, FORGE Canada Consortium, and Care4Rare Canada Consortium. Utility of Whole-Exome Sequencing for Those near the End of the Diagnostic Odyssey: Time to Address Gaps in Care. Clin. Genet..

[4] Amberger J.S., Bocchini C.A., Schiettecatte F., Scott A.F., Hamosh A. (2015). Omim.Org: Online Mendelian Inheritance in Man (Omim^®^), an Online Catalog of Human Genes and Genetic Disorders. Nucleic Acids Res..

[5] Posey J.E. (2019). Genome Sequencing and Implications for Rare Disorders. Orphanet. J. Rare Dis..

[6] Smedley D., Smith K.R., Martin A., Thomas E.A., McDonagh E.M., Cipriani V., Ellingford J.M., Arno G., Tucci A., Vandrovcova J. (2021). 100,000 Genomes Pilot on Rare-Disease Diagnosis in Health Care—Preliminary Report. N. Engl. J. Med..

[7] McInerney-Leo A.M., Duncan E.L. (2021). Massively Parallel Sequencing for Rare Genetic Disorders: Potential and Pitfalls. Front. Endocrinol..

[8] Poon K.-S., Tan K.M.-L. (2021). Reclassification of Whole Exome Sequencing-derived Genetic Variants in Pendred Syndrome with ACMG/AMP Standards. Glob. Med Genet..

[9] De La Vega F.M., Chowdhury S., Moore B., Frise E., McCarthy J., Hernandez E.J., Wong T., James K., Guidugli L., Agrawal P.B. (2021). Artificial Intelligence Enables Comprehensive Genome Interpretation and Nomination of Candidate Diagnoses for Rare Genetic Diseases. Genome. Med..

[10] Matalonga L., Hernández-Ferrer C., Piscia D., Schüle R., Synofzik M., Töpf A., Vissers L.E.L.M., de Voer R., Tonda R., Laurie S. (2021). Solving Patients with Rare Diseases through Programmatic Reanalysis of Genome-Phenome Data. Eur. J. Hum. Genet..

[11] Salfati E.L., Spencer E., Topol S.E., Muse E.D., Rueda M., Lucas J.R., Wagner G.N., Campman S., Topol E.J., Torkamani A. (2019). Re-analysis of whole-exome sequencing data uncovers novel diagnostic variants and improves molecular diagnostic yields for sudden death and idiopathic diseases. Genome Med..

[12] Adzhubei I., Jordan D.M., Sunyaev S.R. (2013). Predicting Functional Effect of Human Missense Mutations Using Polyphen-2. Curr. Protoc. Hum. Genet..

[13] Rentzsch P., Witten D., Cooper G.M., Shendure J., Kircher M. (2019). Cadd: Predicting the Deleteriousness of Variants Throughout the Human Genome. Nucleic Acids Res..

[14] Nicora G., Zucca S., Limongelli I., Bellazzi R., Magni P. (2022). A Machine Learning Approach Based on Acmg/Amp Guidelines for Genomic Variant Classification and Prioritization. Sci. Rep..

[15] Hoffman-Andrews L. (2018). The Known Unknown: The Challenges of Genetic Variants of Uncertain Significance in Clinical Practice. J. Law Biosci..

[16] Anna A., Monika G. (2018). Splicing Mutations in Human Genetic Disorders: Examples, Detection, and Confirmation. J. Appl. Genet..

[17] Evans H.J. (1988). Mutation as a Cause of Genetic Disease. Philos. Trans. R. Soc. Lond. Ser. B Biol. Sci..

[18] de Ligt J., Veltman J.A., Vissers L.E. (2013). Point Mutations as a Source of De Novo Genetic Disease. Curr. Opin. Genet. Dev..

[19] Rahit K.M., Tarailo-Graovac M. (2020). Genetic Modifiers and Rare Mendelian Disease. Genes.

[20] Schaefer J., Lehne M., Schepers J., Prasser F., Thun S. (2020). The Use of Machine Learning in Rare Diseases: A Scoping Review. Orphanet J. Rare Dis..

[21] Sánchez Fernández I., Yang E., Calvachi P., Amengual-Gual M., Wu J.Y., Krueger D., Northrup H., Bebin M.E., Sahin M., Yu K.H. (2020). Deep Learning in Rare Disease. Detection of Tubers in Tuberous Sclerosis Complex. PLoS ONE.

[22] Ai Driving Breakthroughs on Rare Diseases. https://nationalpress.org/topic/ai-driving-breakthroughs-on-rare-diseases/.

[23] Decherchi S., Pedrini E., Mordenti M., Cavalli A., Sangiorgi L. (2021). Opportunities and Challenges for Machine Learning in Rare Diseases. Front. Med..

[24] Fernandez-Marmiesse A., Gouveia S., Couce M.L. (2018). Ngs Technologies as a Turning Point in Rare Disease Research, Diagnosis and Treatment. Curr. Med. Chem..

[25] Ensemble Methods: Bagging, Boosting and Stacking. https://towardsdatascience.com/ensemble-methods-bagging-boosting-and-stacking-c9214a10a205.

[26] Support Vector Machines: A Simple Explanation—Kdnuggets. https://www.kdnuggets.com/2016/07/support-vector-machines-simple-explanation.html.

[27] What Are Neural Networks?. https://www.ibm.com/cloud/learn/neural-networks.

[28] https://Www.Pharmasug.Org/Proceedings/2019/St/Pharmasug-2019-St-325.Pdf.

[29] Mitani A.A., Haneuse S. (2020). Small Data Challenges of Studying Rare Diseases. JAMA Netw. Open.

[30] Three Rare Disease Diagnostic Opportunities for Ai and Machine Learning. https://insights.axtria.com/blog/three-rare-disease-diagnoses-opportunities-for-ai/ml-artificial-intelligence-and-machine-learning.

[31] Ioannidis N.M., Rothstein J.H., Pejaver V., Middha S., McDonnell S.K., Baheti S., Musolf A., Li Q., Holzinger E., Karyadi D. (2016). Revel: An Ensemble Method for Predicting the Pathogenicity of Rare Missense Variants. Am. J. Hum. Genet..

[32] Gunning A.C., Fryer V., Fasham J., Crosby A.H., Ellard S., Baple E.L., Wright C.F. (2021). Assessing Performance of Pathogenicity Predictors Using Clinically Relevant Variant Datasets. J. Med. Genet..

[33] Munshani S., Ibrahim E.Y., Domenicano I., Ehrlich B.E. (2021). The Impact of Mutations in Wolframin on Psychiatric Disorders. Front. Pediatrics.

[34] Boudellioua I., Kulmanov M., Schofield P.N., Gkoutos G.V., Hoehndorf R. (2018). Oligopvp: Phenotype-Driven Analysis of Individual Genomic Information to Prioritize Oligogenic Disease Variants. Sci. Rep..

[35] Rao A., Vg S., Joseph T., Kotte S., Sivadasan N., Srinivasan R. (2018). Phenotype-Driven Gene Prioritization for Rare Diseases Using Graph Convolution on Heterogeneous Networks. BMC Med. Genom..

[36] Díaz-Santiago E., Jabato F.M., Rojano E., Seoane P., Pazos F., Perkins J.R., Ranea J.A.G. (2020). Phenotype-Genotype Comorbidity Analysis of Patients with Rare Disorders Provides Insight into Their Pathological and Molecular Bases. PLoS Genet..

[37] Jia J., Wang R., An Z., Guo Y., Ni X., Shi T. (2018). Rdad: A Machine Learning System to Support Phenotype-Based Rare Disease Diagnosis. Front. Genet..

[38] Qi H., Zhang H., Zhao Y., Chen C., Long J.J., Chung W.K., Guan Y., Shen Y. (2021). Mvp Predicts the Pathogenicity of Missense Variants by Deep Learning. Nat. Commun..

[39] Yandell M., Huff C., Hu H., Singleton M., Moore B., Xing J., Jorde L.B., Reese M.G. (2011). A Probabilistic Disease-Gene Finder for Personal Genomes. Genome Res..

[40] Singleton M.V., Guthery S.L., Voelkerding K.V., Chen K., Kennedy B., Margraf R.L., Durtschi J., Eilbeck K., Reese M.G., Jorde L.B. (2014). Phevor Combines Multiple Biomedical Ontologies for Accurate Identification of Disease-Causing Alleles in Single Individuals and Small Nuclear Families. Am. J. Hum. Genet..

[41] Robinson P.N., Köhler S., Oellrich A., Wang K., Mungall C.J., Lewis S.E., Washington N., Bauer S., Seelow D., Krawitz P. (2014). Improved Exome Prioritization of Disease Genes through Cross-Species Phenotype Comparison. Genome Res..

[42] Https://Fabricgenomics.Com/Wp-Content/Uploads/2021/09/202011-Fabric-Gem-Data-Sheet-Final.Pdf.

[43] Lek M., Karczewski K.J., Minikel E.V., Samocha K.E., Banks E., Fennell T., O’Donnell-Luria A.H., Ware J.S., Hill A.J., Cummings B.B. (2016). Analysis of Protein-Coding Genetic Variation in 60,706 Humans. Nature.

[44] Hoskinson D.C., Dubuc A.M., Mason-Suares H. (2017). The Current State of Clinical Interpretation of Sequence Variants. Curr. Opin. Genet. Dev..

[45] Federici G., Soddu S. (2020). Variants of Uncertain Significance in the Era of High-Throughput Genome Sequencing: A Lesson from Breast and Ovary Cancers. J. Exp. Clin. Cancer Res..

[46] Schubach M., Re M., Robinson P.N., Valentini G. (2017). Imbalance-Aware Machine Learning for Predicting Rare and Common Disease-Associated Non-Coding Variants. Sci. Rep..

[47] Kircher M., Witten D.M., Jain P., O’Roak B.J., Cooper G.M., Shendure J. (2014). A General Framework for Estimating the Relative Pathogenicity of Human Genetic Variants. Nat. Genet..

[48] Zaucha J., Heinzinger M., Tarnovskaya S., Rost B., Frishman D. (2020). Family-Specific Analysis of Variant Pathogenicity Prediction Tools. NAR Genom. Bioinform..

[49] Iancu I.F., Avila-Fernandez A., Arteche A., Trujillo-Tiebas M.J., Riveiro-Alvarez R., Almoguera B., Martin-Merida I., Del Pozo-Valero M., Perea-Romero I., Ayuso C. (2021). Prioritizing Variants of Uncertain Significance for Reclassification Using a Rule-Based Algorithm in Inherited Retinal Dystrophies. NPJ Genom. Med..

[50] Kim S., Jhong J.H., Lee J., Koo J.Y. (2017). Meta-Analytic Support Vector Machine for Integrating Multiple Omics Data. BioData Min..

[51] Zeng Z., Bromberg Y. (2019). Predicting Functional Effects of Synonymous Variants: A Systematic Review and Perspectives. Front. Genet..

[52] Jaganathan K., Panagiotopoulou S.K., McRae J.F., Darbandi S.F., Knowles D., Li Y.I., Kosmicki J.A., Arbelaez J., Cui W., Schwartz G.B. (2019). Predicting Splicing from Primary Sequence with Deep Learning. Cell.

[53] Lord J., Baralle D. (2021). Splicing in the Diagnosis of Rare Disease: Advances and Challenges. Front. Genet..

[54] Cheng J., Nguyen T.Y.D., Cygan K.J., Çelik M.H., Fairbrother W.G., Avsec Ž., Gagneur J. (2019). Mmsplice: Modular Modeling Improves the Predictions of Genetic Variant Effects on Splicing. Genome Biol..

[55] Rentzsch P., Schubach M., Shendure J., Kircher M. (2021). Cadd-Splice-Improving Genome-Wide Variant Effect Prediction Using Deep Learning-Derived Splice Scores. Genome Med..

[56] Darling A.L., Uversky V.N. (2018). Intrinsic Disorder and Posttranslational Modifications: The Darker Side of the Biological Dark Matter. Front. Genet..

[57] Brooks P.J., Tagle D.A., Groft S. (2014). Expanding Rare Disease Drug Trials Based on Shared Molecular Etiology. Nat. Biotechnol..

[58] Li G., Panday S.K., Alexov E. (2021). Saafec-Seq: A Sequence-Based Method for Predicting the Effect of Single Point Mutations on Protein Thermodynamic Stability. Int. J. Mol. Sci..

[59] Caragea C., Sinapov J., Silvescu A., Dobbs D., Honavar V. (2007). Glycosylation Site Prediction Using Ensembles of Support Vector Machine Classifiers. BMC Bioinform..

[60] Capriotti E., Fariselli P., Casadio R. (2005). I-Mutant2.0: Predicting Stability Changes Upon Mutation from the Protein Sequence or Structure. Nucleic Acids Res..

[61] Chen C.W., Lin J., Chu Y.W. (2013). Istable: Off-the-Shelf Predictor Integration for Predicting Protein Stability Changes. BMC Bioinform..

[62] Browne C., Timson D.J. (2015). In Silico Prediction of the Effects of Mutations in the Human Mevalonate Kinase Gene: Towards a Predictive Framework for Mevalonate Kinase Deficiency. Ann. Hum. Genet..

[63] Brasil S., Pascoal C., Francisco R., Dos Reis Ferreira V., Videira P.A., Valadão A.G. (2019). Artificial Intelligence (Ai) in Rare Diseases: Is the Future Brighter?. Genes.

[64] Kousi M., Katsanis N. (2015). Genetic Modifiers and Oligogenic Inheritance. Cold Spring Harb. Perspect. Med..

[65] Mukherjee S., Cogan J.D., Newman J.H., Phillips J.A., Hamid R., Meiler J., Capra J.A. (2021). Identifying Digenic Disease Genes Via Machine Learning in the Undiagnosed Diseases Network. Am. J. Hum. Genet..

[66] Gazzo A.M., Daneels D., Cilia E., Bonduelle M., Abramowicz M., Van Dooren S., Smits G., Lenaerts T. (2016). Dida: A Curated and Annotated Digenic Diseases Database. Nucleic Acids Res..

[67] Papadimitriou S., Gazzo A., Versbraegen N., Nachtegael C., Aerts J., Moreau Y., Van Dooren S., Nowé A., Smits G., Lenaerts T. (2019). Predicting Disease-Causing Variant Combinations. Proc. Natl. Acad. Sci. USA.

[68] Dallali H., Kheriji N., Kammoun W., Mrad M., Soltani M., Trabelsi H., Hamdi W., Bahlous A., Ben Ahmed M., Mahjoub F. (2021). Multiallelic Rare Variants in Bbs Genes Support an Oligogenic Ciliopathy in a Non-Obese Juvenile-Onset Syndromic Diabetic Patient: A Case Report. Front. Genet..

[69] 100,000 Genomes Project 2021 Update: Rare Disease—Genomics Education Programme. https://www.genomicseducation.hee.nhs.uk/blog/100000-genomes-project-2021-update-rare-disease/.

[70] Khost D.E., Eickbush D.G., Larracuente A.M. (2017). Single-Molecule Sequencing Resolves the Detailed Structure of Complex Satellite DNA Loci in Drosophila Melanogaster. Genome Res..

[71] Ameur A., Kloosterman W.P., Hestand M.S. (2019). Single-Molecule Sequencing: Towards Clinical Applications. Trends Biotechnol..

[72] Luo R., Sedlazeck F.J., Lam T.W., Schatz M.C. (2019). A Multi-Task Convolutional Deep Neural Network for Variant Calling in Single Molecule Sequencing. Nat. Commun..

[73] Yin Q., Wang Y., Guan J., Ji G. (2022). Sciae: An Integrative Autoencoder-Based Ensemble Classification Framework for Single-Cell Rna-Seq Data. Brief. Bioinform..

[74] Li H., Brouwer C.R., Luo W. (2022). A Universal Deep Neural Network for in-Depth Cleaning of Single-Cell Rna-Seq Data. Nat. Commun..

[75] Wang Y., Zhao H. (2022). Non-Linear Archetypal Analysis of Single-Cell Rna-Seq Data by Deep Autoencoders. PLoS Comput. Biol..

[76] Pratella D., Ait-El-Mkadem Saadi S., Bannwarth S., Paquis-Fluckinger V., Bottini S. (2021). A Survey of Autoencoder Algorithms to Pave the Diagnosis of Rare Diseases. Int. J. Mol. Sci..

[77] Ergin S., Kherad N., Alagoz M. (2022). RNA sequencing and its applications in cancer and rare diseases. Mol. Biol. Rep..

[78] Komlósi K., Gyenesei A., Bene J. (2022). Editorial: Copy Number Variation in Rare Disorders. Front. Genet..

[79] Requena F., Abdallah H.H., García A., Nitschké P., Romana S., Malan V., Rausell A. (2021). Cnvxplorer: A Web Tool to Assist Clinical Interpretation of Cnvs in Rare Disease Patients. Nucleic Acids Res..

[80] Gabrielaite M., Torp M.H., Rasmussen M.S., Andreu-Sánchez S., Vieira F.G., Pedersen C.B., Kinalis S., Madsen M.B., Kodama M., Demircan G.S. (2021). A Comparison of Tools for Copy-Number Variation Detection in Germline Whole Exome and Whole Genome Sequencing Data. Cancers.

[81] Li Y.R., Glessner J.T., Coe B.P., Li J., Mohebnasab M., Chang X., Connolly J., Kao C., Wei Z., Bradfield J. (2020). Rare Copy Number Variants in over 100,000 European Ancestry Subjects Reveal Multiple Disease Associations. Nat. Commun..

[82] Sharo A.G., Hu Z., Sunyaev S.R., Brenner S.E. (2022). Strvctvre: A Supervised Learning Method to Predict the Pathogenicity of Human Genome Structural Variants. Am. J. Hum. Genet..

[83] Bhattacharya S., Barseghyan H., Délot E.C., Vilain E. (2021). Nanotator: A Tool for Enhanced Annotation of Genomic Structural Variants. BMC Genom..

[84] Zhang L., Shi J., Ouyang J., Zhang R., Tao Y., Yuan D., Lv C., Wang R., Ning B., Roberts R. (2021). X-Cnv: Genome-Wide Prediction of the Pathogenicity of Copy Number Variations. Genome Med..

[85] Schlieben L.D., Prokisch H., Yépez V.A. (2021). How Machine Learning and Statistical Models Advance Molecular Diagnostics of Rare Disorders Via Analysis of Rna Sequencing Data. Front. Mol. Biosci..

[86] Taroni J.N., Grayson P.C., Hu Q., Eddy S., Kretzler M., Merkel P.A., Greene C.S. (2019). Multiplier: A Transfer Learning Framework for Transcriptomics Reveals Systemic Features of Rare Disease. Cell Syst..

[87] Kerr K., McAneney H., Smyth L.J., Bailie C., McKee S., McKnight A.J. (2020). A Scoping Review and Proposed Workflow for Multi-Omic Rare Disease Research. Orphanet J. Rare Dis..

[88] Labory J., Fierville M., Ait-El-Mkadem S., Bannwarth S., Paquis-Flucklinger V., Bottini S. (2020). Multi-Omics Approaches to Improve Mitochondrial Disease Diagnosis: Challenges, Advances, and Perspectives. Front. Mol. Biosci..

[89] Liu X., Yang Z., Lin H., Simmons M., Lu Z. (2017). Dignifi: Discovering Causative Genes for Orphan Diseases Using Protein-Protein Interaction Networks. BMC Syst. Biol..

[90] Zhu C., Kushwaha A., Berman K., Jegga A.G. (2012). A Vertex Similarity-Based Framework to Discover and Rank Orphan Disease-Related Genes. BMC Syst. Biol..

[91] Kodra Y., Weinbach J., Posada-de-la-Paz M., Coi A., Lemonnier S.L., van Enckevort D., Roos M., Jacobsen A., Cornet R., Ahmed S.F. (2018). Recommendations for Improving the Quality of Rare Disease Registries. Int. J. Environ. Res. Public Health.

